# No degradation of temperature-mediated phenotypic plasticity in *Drosophila melanogaster* after more than 275 generations of artificial selection on body-size.

**DOI:** 10.17912/micropub.biology.001468

**Published:** 2025-04-04

**Authors:** Yvonne L. Ofodile, Judith Appenteng, Mubeen Jaffri, Ian Dworkin, Andrew D. Stewart

**Affiliations:** 1 Biology, Canisius University, Buffalo, New York, United States; 2 Biology, McMaster University, Hamilton, Ontario, Canada

## Abstract

Body size is a fundamental trait that shapes a species' development and evolution. Importantly, body size can also be affected by environmental variables, especially development temperature. Here we measure phenotypic plasticity in a series of lineages that had experienced artificial selection on body size for over 275 generations. We found, despite substantial changes in overall size and sexual size dimorphism, only modest effects on developmental plasticity. Still, there were some significant, changes in the sex specific slopes of the relationship between size and rearing temperature, largely due to a reduction in plasticity in the treatment selected for small body size.

**Figure 1. Evolutionary and phenotypically plastic changes in thorax size in Drosophila:**
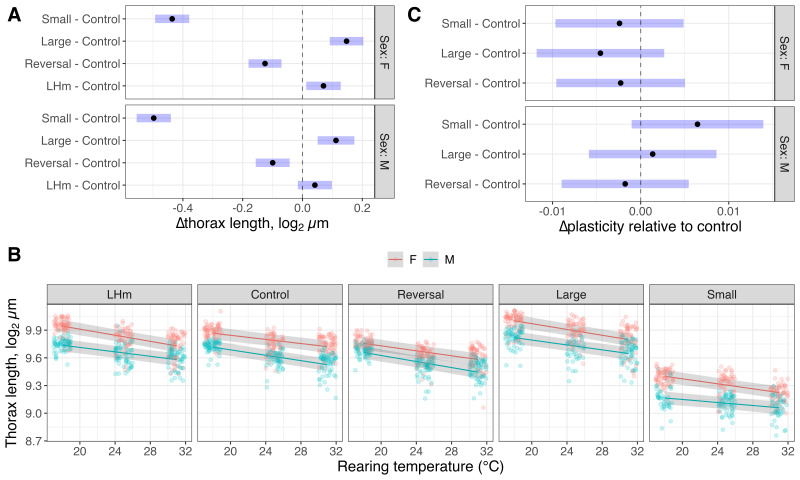
(A) Differences in Thorax Length by Sex: *log _2_* transformed expected marginal means of thorax length, in µm, for each lineage (Large, Small, Reversed, and LH _M_ ) minus the expected marginal mean of the Control lineage (black dots), with 95% confidence interval (blue bars), are shown for each sex. All displayed values are from flies reared at the standard culturing temperature (25°C). Positive values indicate treatments with larger mean thorax lengths than controls, while negative values indicate smaller average lengths. (B) Thorax Length by Temperature: log _2_ transformed thorax lengths, in µm (open circles), for each rearing temperature (18°C, 25°C, & 31°C) within each lineage (Large, Small, Reversed, Control, and LH _M_ ), are shown for males (blue) and females (red). Estimated fits, with 95% confidence bands (grey shaded area). (C) Changes in Phenotypic Plasticity by Sex and treatment: Predicted changes in slopes for each lineage (Large, Small, and Reversed) compared with the control lineage (black dots), with 95% confidence interval (blue bars), are shown for each sex. Model fit and contrasts using log _2_ transformed thorax length (µm) to facilitate proportional comparisons given the substantial differences in mean trait size across evolutionary treatments.

## Description

The evolution of body size is one of the most striking and ubiquitous features of multicellular life, in particular for plants and animals. This variation is not only observed among different species, but differs among populations within species, among individuals within populations, and in many species via sexual size dimorphism (Blanckenhorn, 2000; Maurer et al., 1992; Rohner et al., 2018). This variation is not surprising, as traits that relate or contribute to body size are often functionally important, have direct impacts on organismal performance and fitness, and, in particular, mediate multiple fitness components (Brown et al., 1993). While “size” itself is a composite trait, proxy traits for overall size often have high heritabilities (Robertson & Reeve, 1952) in many (though not all) species, possibly due to large mutational target size (Carreira et al., 2009). Given the potential for strong selection and high heritabilities, it is not surprising to see considerable, and often rapid, evolution of size related traits within species. For instance, in *Drosophila melanogaster* , there are consistent ecological patterns of size related evolution associated with altitudinal, latitudinal, and seasonal variation (Fabian et al., 2015; Gilchrist & Partridge, 1999; Huey et al., 2000; James et al., 1997; Klepsatel et al., 2014; Lack et al., 2016; Önder & Aksoy, 2022; Pitchers et al., 2013).

Importantly though, variation in size is jointly influenced, often to a substantial degree, by environmental variables. In *D. melanogaster* , many factors including larval nutrition and density, rearing temperature, and O _2_ concentration all induce a phenotypically plastic response in body size, often in complex ways (Alpatov, 1930; Bitner–Mathé & Klaczko, 1999; Czarnoleski et al., 2023; Frazier et al., 2001; Nijhout et al., 2014; Peck & Maddrell, 2005; Powell et al., 2010; Shingleton et al., 2009). Phenotypic plasticity in size, mediated by rearing temperature, has been broadly studied in many different evolutionary contexts. Several studies have demonstrated an association between this temperature-mediated plasticity and patterns observed in nature (Bitner–Mathé & Klaczko, 1999; Debat et al., 2009; French et al., 1998; Partridge & French, 1996). This has been interpreted by some authors to suggest that this represents a form of adaptive plasticity. On the other hand, the generally observed pattern of increasing body size with decreasing rearing temperature, may represent an ancestral state, and potentially even representing some physiological requirements for growth (Powell et al., 2010). However, it is known that there is considerable genetic variation for thermal plasticity within and between populations as well (Lafuente et al., 2018; Scheiner & Lyman, 1991). This has led us to ask two main questions. First, to what degree is thermal plasticity a function of the range of body sizes of *D. melanogaster* , and, second, how might thermal plasticity change (if at all), in the face of strong, persistent directional selection for body size? In this study we use a long-term artificial selection study of body size in *D. melanogaster* to examine the degree to which temperature mediated plasticity in body size changed after 287 generations of artificial selection for either increased or decreased body size (Audet et al., 2024; Stewart & Rice, 2018; Turner et al., 2011). We demonstrate that, despite a substantial response to changes in overall size among the lineages, proportional changes in size in response to temperature mediated developmental plasticity are modest.

After 287 generations of artificial selection, patterns of body size differences corresponded with the relevant selection regime, and consistent with observations at earlier time points (Stewart & Rice, 2018; Turner et al., 2011). Lineages selected for small body size have, on average, a 29% (95% CI: 26%-32%) reduction in mean thorax length, compared with corresponding evolutionary controls in males ( Figure 1A ). The reduction seen in females was similar, with a reduction of 26% (95% CI: 23-29%). Artificial selection for increased size resulted in an average increase of 8% in males (95%CI: of 4%-13% increase) and 11% in females (95% CI: 6-15%), compared to the corresponding control treatment for each sex. Conversely, there were only small differences in body size between the LH _M_ “ancestral” lineage (from which all body size lineages were founded) and the experimental controls. LH _M_ males were, on average, 3% larger (95% CI :1% decrease to 7% increase), and females were 5% larger (95% CI: 1-9% increase), than the control treatments.

Despite substantial changes in overall size and sexual size dimorphism after 287 generations of consistently applied artificial selection in a common environment, the effects on developmental plasticity for size are modest ( Figure 1B ). Despite this, there are some subtle, but significant, changes in the sex specific slopes of the relationship between size and rearing temperature ( Figure 1C ). Assuming a linear relationship, in the control treatment, the expected reduction in thorax length corresponds to 6.9 µm/°C (95% CI: 4.5 – 9.3 µm/°C) in females, representing about a 0.7% reduction in thorax length per °C increase in rearing temperature (9.6% over 13°C). For control males, our estimates are of a decrease of 7.9 µm/°C (95% CI: 5.5 – 10.3 µm/°C), corresponding to 0.95% reduction per °C (12.3% reduction over 13°C). The ANOVA shows evidence for an interaction between evolutionary treatment, sex and rearing environment (χ ^2^ = 22.4, df = 4, *p* = 0.00017). This is in large part due to a marked reduction in plasticity observed in the treatment artificially selected concordantly for small body size, where we see only a ~0.55% reduction in thorax length per °C (7.1% over the 13°C range) in males. We see limited evidence for changes in temperature mediated developmental plasticity for size in all other treatments ( Figure 1C ).

More than 275 generations of persistent, strong directional selection resulted in substantial evolution of body size ( Figure 1A ), yet only modest impacts on temperature mediated plasticity in size ( Figure 1B & C). Previous studies in *Drosophila* have demonstrated the persistence of temperature-mediated plasticity on body size (Alpatov, 1930; McCabe & Partridge, 1997), although some changes in plasticity have been observed (Noach et al., 1997). There is also considerable genetic variation for the degree of temperature-mediated plasticity, and this plasticity has also been shown to be able to evolve when selected upon (Scheiner & Lyman, 1991). In the context of previous results and our current findings, this suggests several “things”. First, it is unlikely that the observed pattern of temperature mediated plasticity is mediated solely due to adaptive plasticity. If this was the case, then we would expect to observe some degradation of this plasticity in all lineages, which have been reared under constant environmental conditions for more than 500 total generations (lab domestication of LH _M_ , plus artificial selection). Secondly, our results potentially suggest that there may be, as of yet unknown, necessary physiological or developmental mechanisms that pleiotropically, due to potential impacts on fitness, result in the general pattern of increased body size with decreasing rearing temperature (Powell et al., 2010). Finally, our results demonstrate that the majority of allelic variants contributing to evolution of body size (at least in the ancestral LH _M_ population) do not have substantial pleiotropic impacts on temperature plasticity. It is important to note that this is not simply the result of relaxed selection in lab conditions (in general), as LH _M_ has been shown to have adaptively evolved with respect to numerous fitness-proximal traits (Ruzicka et al., 2019), and lab domestication in *Drosophila* often involves strong natural selection (Hoffmann & Ross, 2018). Further work determining the physiological and/or developmental mechanisms that may pleiotropically influence and maintain this plasticity is clearly important.

There are several caveats and limitations to this study. First, and despite its wide use, it could be that the wild source population used to found LH _M_ (and ultimately the artificial selection lineages) was unusual in some way compared to other natural populations. However, given that these populations recapitulate ancestral patterns of this plasticity, and was founded with 400 non-virgin females (Rice et al., 2005) from the wild, this seems unlikely. It is also important to note that only a single lineage (of the two available) was used for each of the artificially selected treatments. That said, all lineages we evaluated maintained qualitatively similar patterns of temperature mediated plasticity, it is unlikely that sampling from the additional available replicate lineages would substantially alter the results, nor our inferences. Importantly, we only measured a single trait, thorax length, while selection was actually based on a sieving process, which selects on a complex cross-sectional area. It is known that the response to selection has produced trait-specific magnitudes of response (Turner 2011, Audet et. al. 2024), though with an overall consistent effect based on this artificial selection regime. Future experiments should examine multiple traits (e.g., thorax length, femur length, wing size, etc.), as temperature mediated plasticity for size varies among traits (Shingleton et al., 2009). Lastly, it is possible that body size plasticity has a sufficiently small mutational target size that an insufficient number of mutations have occurred that would disrupt this thermal plasticity in “only” 287 generations. Further examination of these populations at later time points during the ongoing artificial selection experiment would help to address this possibility.

## Methods

*Base LH _M_ population*

*D. melanogaster* artificial selection lineages were all derived from LH _M_ , a large, outbred population. At the start of the body size experiment, LH _M_ had adapted to laboratory conditions for 370 discrete, two-week generations. LH _M_ is reared at 25°C on a 12/12 light/dark cycle. During each generation of culturing, flies are reared in three consecutive sets of vials. In the first set (Day-0), eggs are laid in 56 'juvenile competition' vials. Eggs are manually culled to a density of 150-200 per vial. The resulting progeny remain in juvenile competition vials for the larval, pupal, and early adult stages of life. Later (Day-12), adult flies are mixed between juvenile competition vials and 1792 adults are randomly selected (under brief CO _2_ anesthesia). Adults are transferred to a second set of 56 ‘adult competition' vials (16 pairs/vial) and remain for approximately 2 days. During this time, females compete for a limited supply (10 mg) of live-yeast, a limiting resource that strongly affects a female's fecundity (Carreira et al., 2009), and males compete for fertilizations among females. After adult competition, all adult flies are transferred, without anesthesia, to a third set of 56 vials (without live-yeast) for 18 hours of ‘oviposition.' After oviposition, eggs are reduced to 150-200 per vial (~9800 eggs) as juvenile competition vials of the next generation. Detailed descriptions of the LH _M_ population and culturing protocols are described elsewhere (Rice et al., 2005). The protocols used during this study (described below) are designed to match, as closely as possible, the culturing protocol of the LH _M_ population.

*Size-Selected Populations*

In addition to LH _M_ , this study utilized four lineages of flies from an artificial selection experiment on total body-size. These populations were simultaneously generated from the LH _M_ base population, then maintained independently since their founding. All populations consisted of 10 vials, with 16 pairs of adult flies per vial. The timing of culturing events and culture conditions (i.e. larval and adult densities, yeast levels, etc.) matched that of LH _M_ , with the lone exception being how adult flies were chosen to propagate subsequent generations. In the small lineage (S), on day 12 of their life-cycle, only the smallest 160 males and 160 females were selected, placed in their 10 adult competition vials, then allowed to breed. Similarly, in the large lineage (L), only the largest 160 males and 160 females were retained each generation. In the Experimental Dimorphism Reversal lineage (RE), the population experienced sexually discordant selection, where only the largest 160 males and the smallest 160 females were selected for breeding. Finally, the control lineage (C) was exposed to the same body size selection protocols as the various selection lines, but no artificial body-size selection was imposed; instead, after sieving, all individuals were pooled and 160 males and 160 females were randomly chosen from the population to found the next generation. As with LH _M_ , after selection, males and females underwent two days of adult competition on limited yeast, followed by 18 hours of yeast-free oviposition. After oviposition, eggs were manually culled to 150-200/vial. Thus, each generation produced approximately 1,500-2,000 adult flies, per treatment, per generation. For a full description of the specific size-selection protocols, see (Stewart & Rice, 2018). While the full set of Stewart & Rice study includes two replicate lineages within each experimental treatment ( *i.e.* S1 & S2, L1 & L2, etc.), due to limitations in available personnel, only one lineage per experimental treatment was assayed here. At the time of this study, the size selection lineages had been continually selected for 287 generations.

*Thermal Plasticity Experimental design*

We measured the body size of each of the four artificial selection lineages (C, RE, L & S), along with the ancestral population (LH _M_ ) that these lineages were founded from, at three different temperatures: 18°C, 25°C, 31°C, with 25°C being the culturing temperature to which the flies were adapted. For each temperature x lineage combination, three replicate vials were created by having a large number of adult females (50+) oviposit on disks of media for a period of 18 hours. After oviposition, 180 eggs were counted and transferred to each of nine standard food vials. This is the normal egg density of both the ancestral population and the selection lineages from which they were sampled. Vials with the eggs were then placed in incubators, set at the appropriate constant temperature (18°C, 25°C, or 31°C), with 12:12 light:dark cycles. Flies were allowed to develop through larval stages, pupation, and adult eclosion. Since development time changes with temperature, we standardized the post-eclosion age of adult flies for subsequent measurements. To do this, adult flies were collected and pooled across the three vials, per temperature x lineage, daily, making sure keep offspring of each size and temperature treatment separate. These flies were then allowed to mature on fresh media for an additional 24-hour period before being transferred to a -20°C freezer for preservation until images could be taken of individual flies. Collections for all vials commenced the first day that any vial contained adult flies (Day 8) and continued until vials no longer produced adult offspring (Day 28). This experimental procedure was replicated two sequential blocks, generating a total of 90 vials (5 lineages X 3 vials X 3 temperatures X 2 replicate blocks).

*Thorax Measurements*

From each vial of preserved adults, where possible, 10 males and 10 females were randomly selected for measuring. As a proxy for body size, we measured thorax length collected from each lineage. Thorax length, was measured as the distance between posterior tip of the scutellum and the most anterior humoral bristl e (see Partridge et al, 1999; Shingleton, 2009). All measurements were done using images taken from an Accu-Scope stereo-microscope at 45X magnification, with a Leica EC3 digital camera. Image analysis was performed using NIH ImageJ (version 1.49r). In total, 802 males and 841 females were measured.

*Data modeling*

We fit a linear mixed model, with log _2_ transformed adult thorax length (µm) as a response. The predictors in the model including sex, evolutionary treatment, developmental rearing temperature and their interactions. Random effect for replicate block and independent random effect for vials nested within evolutionary lineage, temperature and block were included. Models were fit using lmer in lme4 (v1.1-28), (Bates et al., 2015) . Estimated coefficients and confidence intervals were extracted using emmeans (v1.7.3) (Lenth, 2024). Interaction contrasts from the model to assess changes in slopes between thorax length and rearing temperature across evolutionary treatments were also conducted using emmeans. Predicted values and their confidence intervals were extracted using predictorEffect from the effects library (v4.2-1) (Fox, 2003). Analysis of variance for the mixed model was fit using the Anova function in car (v3.0.12) (Fox & Weisberg, 2019). Plotting was done using ggplot2 (v3.3.5), ggbeeswarm (v0.6.0) and ggdist(3.1.0) (Kay, 2024; Wickham, 2016).

All associated data and scripts can be found on Figshare (dx.doi.org/10.6084/m9.figshare.28692473).

## Reagents

**Table:** 

Strain/Population	Genotype	Available From
LH _M_	*Drosophila melanogaster*	Authors
Body Size Lineages	*Drosophila melanogaster*	Authors
